# Appetite, Metabolism and Hormonal Regulation in Normal Ageing and Dementia

**DOI:** 10.3390/diseases6030066

**Published:** 2018-07-20

**Authors:** Artemissia-Phoebe Nifli

**Affiliations:** Biotechnology, Technological Research Center of Thessaly, 41110 Larissa, Greece; phoebenifli@teilar.gr or nifli@med.uth.gr or f_nifli@hotmail.com; Tel.: +30-694-736-8364

**Keywords:** ageing, dementia, appetite, malnutrition, body composition, growth hormone, adipokines, neurochemistry, attention

## Abstract

Feeding and nutrition follow the growth trajectory of the course of life. The profound physiological changes that human body experiences during ageing affect separate aspects of food intake, from tastant perception to satiety. Concurrent morbidities, such as neurodegeneration, as seen in dementia, and metabolic syndrome, may further shape nutritional behaviours, status and adequacy. In an effort to fill the gap between the exhausting basic research and the actual needs of professionals caring for the exponentially expanding ageing population, the current review addresses major factors relevant to appetite and eating disturbances. Does age alter the perception of food modalities? Is food generally still perceived as alluring and delicious with age? Is there an interplay between ageing, cognitive decline, and malnutrition? What tools can we adopt for proper and timely monitoring? Finally, what anatomical and pathophysiological evidence exists to support a hypothesis of central regulation of metabolic perturbations in normal and accelerated cognitive impairment, and how can we benefit from it in health practice?

## 1. Introduction

Non-cognitive perturbations are frequent in the ageing population [1]. Despite their disparate nature, they are significantly correlated with cognitive impairment, regardless of the establishment of a definite diagnosis [2,3]. The battery of symptoms covers different neuropsychiatric aspects, such as anxiety, depression, apathy, delusions, agitation, irritability or elation, motor behaviours, as well as sleep and eating disturbances. Feeble cognitive function and performance are the most warning signs and key diagnostic factors [4]. However, behavioural and psychological manifestations are the ones undermining caregiving, further affecting the quality of life; their type, severity, and frequency, thus clinical relevance, depend on the age and disease stage [5].

Appetite/eating impairment is one of the most common and intense findings, because of the general decline of physiological systems in the elderly [5,6,7]. The underlying mechanisms may extend from the degeneration of peripheral systems recognizing chemical senses to the central mechanisms orchestrating appetite and hedonic food intake. The cluster is also preeminent among cognitive neurodegenerative diseases, classified under other degenerative diseases of the nervous system (2018 ICD-10-CM, G30-G32), primarily in frontotemporal dementia (FTD), and Alzheimer’s dementia (AD), and less in vascular dementia (VD) [8,9,10,11]. There is a high probability for behavioural and psychological symptoms of dementia (BPSD) to emerge together [1]. Specifically, eating disturbances have been rather related to the behavioural control component of the Neuropsychiatric Inventory (NPI) [12,13]. ApoE4 allele, a risk factor of AD, has a synergistic effect on neuropsychiatric symptoms, mainly depression and apathy, but no relationship with appetite or weight loss [14,15], while decreased appetite or weight is less common in AD patients, with or without depression, than in age-matched depressed controls [16]. Therefore, it could be speculated that the manifestation of impairment in both ageing and neurodegeneration may be linked to discrete molecular, hormonal, or anatomical defects.

The impact of metabolism, as highlighted nowadays [17], has long been suspected, since the initial analysis of BPSD and the development of related tools: weight loss and appetite have been categorized within the “vegetative features” of BPSD, associated with other “rhythmic disturbances”, such as altered sleep pattern [18,19,20,21]. Considering the progress made in the field during the last decades, it would be useful to recapitulate food perception and metabolism during the course of ageing. Accordingly, we will discuss their implications in cognitive function and prosperity in late life, focusing in the two extremes: peripheral sensing and central processing.

## 2. Can We Maintain Sensitivity against Food Modalities over Age?

**Figure 1:**
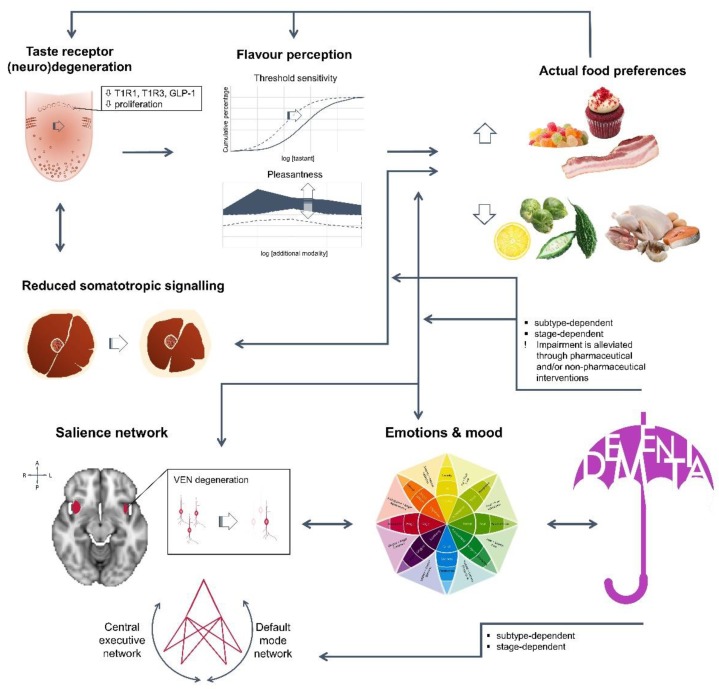
In dementia patients, weight and appetite impairment are due to multiple and reciprocal interconnections among body systems, with cognitive decline adding to the constitutive ageing process, rendering subjects vulnerable to malnutrition and frailty. T1R1: G protein-coupled Taste Receptor type 1 member 1; T1R3: G protein-coupled Taste Receptor type 1 member 3; GLP-1: Glucagon-Like Peptide-1; VEN: Von Economo neurons.

The typical measures of eating disturbances, weight and appetite, arise from profound anatomical, neuronal and metabolic changes, either at peripheral or central level (Figure 1). First, ageing requires sustained mastication for prolonged time, in order for the hardest food to be partitioned to the smallest pieces. In fact, bolus size barely changes, thus bolus particles are larger in those not maintaining natural dentition [22,23]. Perceived inability to mash food has been associated with mild dysgeusia [24], digestive and psychological stress [25], as well as with depression [26]. Second, taste perception is also affected by taste bud receptors’ density and functionality. In humans, a decrease in foliate and circumvallate papillae has been reported over the age of 74 [27], as well as a gradual decrease of fungiform papillae by the age of 40, resulting in significant modification of sensitivity threshold [28]. Similar findings have been reported in adult (18 months old animals, equivalent to a 45-year-old man) and elder male mice (30 months old animals, equivalent to a 70-year-old man) for taste bud size and number of taste cells per bud [29,30]. Early functional behavioural studies have also showed differences between young and elder adults, regarding either taste threshold sensitivity, recognition accuracy or intensity ratings, depending on the protocol [24,31]. Besides age, inter-individual variability, especially against bitter or sour insults [31,32], could be further due to the genetic background [33]. Moreover, intra-variability could be due to the innervation site [24,28,34], in line with the reported differences in bud density and morphology, as well as to additional molecular changes. Recently, down-regulation of taste receptors T1R1 and T1R3A, lying within the foliate papillae, has been shown in patients with no psychogenic taste disorder (65–89 years old) [34]. Similarly, reduced expression of T1R3, and of the taste modulator glucagon-like peptide-1 (GLP-1), was found associated with low sensitivity against sweet stimuli in ageing rats [29,30]. Furthermore, localized low-grade inflammation, through TNFα up-regulation, has been shown to decrease taste cell progenitors and taste bud density in a high-fat diet-induced mice obesity model [35]. Therefore, tastants’ identification is expected to be impaired with age, leading subsequently to behavioural adaptations [25], while reduced sensitivity may also increase the risk of food poisoning or over-exposure to environmentally hazardous chemicals [36]. On the other hand, some nutritional interventions, such as repeated exposure to monosodium glutamate (MSG) [34], may restore receptor levels in the elderly and significantly improve appetite and weight.

## 3. Is Food Still Perceived as Alluring and Delicious with Age?

The amelioration of appetite after increasing tastants’ content accentuates the impact of taste dimensions in the formulation of expectation and memory and specifically that of valence in following a selected regimen even at late age. The slight differences in the slope of perceived intensity as described above, are amplified when taste pleasantness is investigated, either in a bland (e.g., deionized water) or beverage background, with aged individuals rating more satisfying high concentration sucrose and NaCl solutions than the younger ones [31]. However, actual preferences gravitate towards low or subthreshold concentrations regardless of age and are more homogenous in the elderly [37,38]. It is possible that the presence of various tastants in food imparts complex flavours and reduces the need for higher singular stimuli, compensating for the decreased sensitivity. Enhancement of the dominant taste may generate “positive” or “negative” feelings towards the rest of taste qualities, as shown in a food matrix; umami was the only quality that did not receive or cause suppression [39,40]. Accordingly, the addition of MSG was found capable to re-orient meal choices, with no change in total energy uptake in laboratory, residential or hospital settings [41,42]. The optimal concentration of MSG was 0.5–0.6%, quite higher than the one reported in regular Asian umami consumers, but similar to the one reported in the young [43]. Because of the increased palatability, it was also feasible to diminish in parallel the optimal fat, by 30%. All subjects consumed less sweet desserts, and women in particular 4-fold more chicken [41]. Olfactory signals are equally important in the perception of food modalities. Usually, elders may complain about encountering difficulties in everyday life, related to olfactory function [44], and major impairment (odour score < 4 or flavour score < 2) may reach 46% in non-diabetic functional subjects [45]; the lowest the score, the lower their interest in enjoying cooking or eating a wide variety of foods, especially sour, bitter or pungent, and in consuming low-fat milk, and the highest their preference for sweets. Nevertheless, even a small odorant quantity is sufficient to acknowledge satisfaction [32,44], while introducing and increasing in parallel tastants and odorants, may set valence as high as the maximal in the young, and produce linear intensity responses [46]. Subsequently, the manipulation of food components to improve food valence and appetite could be of value in everyday practice.

Comorbidities may also modify perception of food modalities. The higher prevalence of depression in the ageing population could justify the differential response towards olfactory cues as compared to the young [47]. Diabetes mellitus type 2 (DM2) may also work as an independent risk factor for olfactory and cognitive dysfunction, whereas early studies showed a preference for sweet foods, especially in AD and VD [48]. Recently, olfactory impairment was found more common in DM2 patients (*n* = 250, 68–77 years old) with possible cognitive impairment or probable dementia, with Mini Mental State Examination (MMSE) score being negatively associated with HbA1c and serum adiponectin levels [49]. Deficits could be due to neurodegeneration of the olfactory bulb and piriform cortex, as described in T2D Goto–Kakizaki rats, non-reversible by glucose regulating agents such as linagliptin [50]. In line with these findings, University of Pennsylvania Smell Identification Test (UPSIT) scores were found associated with MMSE scores and higher total cerebrospinal fluid (CSF) tau in 160 subjects in a European study, while odour impairment could predict the decline of cognitive function 3 years later [51]. So far, the existing pathoanatomical or molecular evidence linking flavour, metabolic and cognitive impairment is limited, but the field studying the modifications of associative learning with age is growing.

## 4. The Importance of Affect in the Restitution of Appetite Impairment

Neurodegenerative conditions, including ageing and the various dementia subtypes, are the second common cause among patients suffering from smell and taste disorders [52,53]. Subjects usually describe appetite loss, unintended weight loss, malnutrition, and reduced quality of life, while 25% seeks further professional advice [54]. Because of the conversion of neurochemical pathways, the majority of modern interventions focus in the combined perception of flavour, and consider novel modalities that contribute to mouthfeel, such as fat content or viscosity [55]. However, it is not well established whether such input may further shape perceived intensity or valence. The degree of familiarity with cooking procedures and the use of soy condiment Doenjang was found essential for adult Korean subjects (20–64 years old) to give both the traditional type of paste, and the soup made with it, higher pleasantness ratings [56]. Mouthful feelings (e.g., burning, biting, astringent, powdery) though were among the attributes with negative affective impact for both preparations. On the contrary, older Irish adults (74 ± 6 years old), being offered a variety of soups, placed much emphasis on grading product texture, and their preference eased with increased soup thickness across the two experimental levels of trigeminal excitation [57]. In a another study in juice consumers (*n* = 52, 71 ± 7 years old), some followed young adults pattern, highly enjoyed pulpiness, but had lower sensitivity, those with fair sensitivity appreciated more juice sweetness and less bitterness, while for a third sub-group neither attribute justified preference [58]. A more scholarly approach took benefit of displaying 30 food pictures in juxtaposition of 9 positive and 11 negative emotional terms, and their intensity, instead of tasting and a standard Likert scale [59]: participants (*n* = 52, 63–80 years old) chose both types of terms, while ascribed “to like”, “satisfaction”, “interest”, “thrilled” and “serene” the highest intensities, and negatives emotions the lowest. The group comprising the most pleasant foods was less rich in protein, with no significant differences in energy, carbohydrate or lipid content. Some foods generated “frustration”, “guilt” or “surprise”; dry sausage, cream cake, chocolate and fruit tart in women, and turkey cutlet in men. Additionally, “doubt”, “uneasiness”, “indifference” and “disappointment” were more common to the lower quartile of total daily energy intake (*n* = 13; six men and seven women) than the upper (*n* = 13; six men and seven women). Such findings are indicative of the depressive background symptoms in malnourished elder subjects with marginal or deteriorating cognitive impairment [1,6,60]. It is therefore simplistic to confine flavour-related affect within the strongly dislike–strongly like frame, or presume pleasant aliments acquired.

Occasionally, valence may interfere with satiety mechanisms. For example, a sub-group of undernourished hospitalized patients (serum albumin < 35 g/L) opted to consume either the most-preferred supplement (sweeter and with enhanced flavour than the commercially available product), or the less appreciated, during snack time, with a concomitant increase in meal energy uptake, up to 32% [61]. In those encouraged to consume even some small quantities, despite the expressed pleasantness, energy intake was decreased down to 25%. In other cases, dietary deficits or a homeostatic shift may guide taste preference, as seen in older individuals with lower serum albumin and higher blood urea nitrogen (BUN), opting for a soup enriched in casein hydrolysate (1–5% *w*/*v*), regardless of the perceived intensity of amino acid solutions [31].

In case of dementia, eating and swallowing disturbances are profound, in line with the stage of the disorders [10,11]. Peripheral mechanisms or age-related impairment may be present, but not sufficient to justify these symptoms [62]. Proxy informants described how familiar smells and tastes of traditional food awoke pleasant memories and triggered feelings of belonging and joy in patients with dementia [63]. The study took place in three different cultural settings, a geriatric institution in the city of Bergen, Western Norway, a geriatric facility in a Sami town in northern Norway and 4 geriatric facilities in Tshwane, South Africa. “Patients who were ordinarily unable to eat unassisted sat nicely at table and ate without help, and some who usually made a great mess were able to eat without soiling the tablecloth.” Even an “otherwise mute old Sami lady with dementia” commented on how to make and serve savoury pancakes and then fell back in her peace. Therefore, the exploitation of food modalities to improve patient’s life quality would require in depth knowledge of recognition, memory and reward processes.

## 5. Is Energy and Nutrient Imbalance a Hallmark of Ageing?

The typical measures of eating disturbances, weight and appetite, are considered as the final physiological adjustments of the ageing body, and malnutrition, imbalance in a person’s intake of energy and/or nutrients ensues [64]. Obesity is currently the most prevailing type worldwide, with WHO reporting more than 1.9 billion overweight adults, of whom over 650 million obese [65], at least half of the adult population of the Organisation for Economic Co-operation and Development member countries [66]. Elevated body mass index (BMI) is a major risk factor for non-communicable diseases affecting both young and older adults, while it may coexist with the two other types of malnutrition, micronutrient deficiency/excess, or wasting [67,68]. Malnutrition in the elderly may be observed in all three types, and the overall prevalence is estimated higher than that in the general adult population [69]. Thirty-eight percent of community dwelling elderly and 67% of nursing homes residents are malnourished or at risk of malnutrition. During hospitalization or rehabilitation, the percentage may be as high as 86%. Because malnutrition significantly increases morbidity and mortality and compromises the outcome of other underlying conditions and diseases, it was imperative to develop a tool for the monitoring of the condition.

Since the 1990s, Mini Nutritional Assessment^®^ (MNA^®^, Société des Produits Nestlé, S.A., Vevey, Switzerland) has been widely used as a nutrition component of the Comprehensive Geriatric Assessment (CGA) for individuals over the age of 65 [70]. The full version comprises of 18 items, and classifies one as normally nourished, at risk of malnutrition, or malnourished. Neuropsychological problems, dementia and/or depression, are also roughly incorporated using a combined question. A modified version, Self MNA^®^, allows community-dwelling elders to fill the form by themselves [71]. It should be noted that because of the significance of muscle atrophy in malnutrition, regardless of the occurrence of overweight/obesity, sarcopenia is evaluated by measuring mid-arm and calf circumference (CC) in MNA^®^. CC is preferred as a surrogate over BMI, when applying the shorter versions. The proposed cut-off of 31 cm CC has been validated among others in a large study in Spanish territory (*n* = 22,007) [72], and associated with a 2.4 fold increase of risk of malnutrition in women and 2.9 fold in men, across all age groups (65–69, 70–74, 75–79, 80–84, and ≥85 years old). However, detailed body composition analysis using dual-energy X-ray absorptiometry (DEXA) advised for higher cut-offs in both sexes [73,74].

Another concept addressing malnutrition, with predominant clinical value in Geriatrics, is frailty [75,76]. Since 1989, Fried et al. followed two cohorts of 5317 men and women, >65 years old, and estimated unintentional weight loss, exhaustion, weakness, slowness, and activity levels 4 and 7 years later [77]. These criteria proved to work with comorbidities in precipitating incident of disease, hospitalization, falls, disability, and mortality. In 2007, an international consensus group described physical frailty as a clinical state in which there is an increased vulnerability for dependency and/or mortality, when exposed to a stressor [78]. All persons >70 years old, as well as those with significant weight loss (>5%) due to a chronic disease, should be screened for. In younger age (>60 years), the prevalence of pre-frailty, meeting 1 or 2 of the Fried criteria, is estimated around 35–50%, and is higher in women [79]. Poor nutritional status is not significantly correlated with pre-frailty, but 14 of the MNA^®^ components were found associated with frailty after age-adjusted analysis, as well as with malnutrition and the risk of [80]. It has been argued that malnutrition may overlap with frailty, however, frailty is indicative of a negative accelerated prognosis [76]; in malnourished patients, loss of body tissue because of inadequate food intake or increased requirement could be reversed with refeeding, but loss of body tissue because of other inactivity, hormonal, or medical challenges, would not respond to augmentation of protein and energy intake. Indeed, both young (23 ± 1 years old) and older men (68 ± 2 years old) are able to adapt to over- or under-feeding, as shown by total energy expenditure, weight change and respiratory quotients [81]. Older subjects though displayed limited response at the level of resting energy expenditure (REE) and a significantly delayed thermic response to a standard meal. Rather than the expected impact on metabolic rate, researchers identified an effect on appetite during follow up: younger men exhibited spontaneous hypophagia or hyperphagia, in response to over- or under-feeding respectively, while older men did not [82]. Thus, any dietary interventions would be hindered by the reduced metabolic adaptability of the elderly and the apparent shift of satiety. Furthermore, energy intake in community-dwelling elders (>70 years) has been found correlated with wider dietary diversity, translated into fair micronutrient intake, despite the higher prevalence of vitamin E, calcium, and magnesium deficiencies [83]. Accordingly, elders with low BMI consumed limited energy- and micronutrient-dense foods; none met the estimated average requirements for micronutrients, while 44.6% did not met daily protein reference intake. These findings suggest an interplay among weight loss, micronutrient-related malnutrition and appetite, due to physiological changes, either at the chemosensory level, as described above, or hormonal level, leading eventually to anorexia [6,84].

Adherence to dietary guidelines was found associated with greater physical performance and better health status [85], at least for the decade following [86]. To improve nutrition and care in the elderly, Finland, and Australia and New Zealand disclosed relevant guidelines emphasizing the lack of gold standards to diagnose undernutrition, and of consensus for sarcopenia [87,88]. Despite the 4% reduction in muscle mass loss per decade, over the age of 50, the decrease in mitochondrial density and oxidative capacity, bearing a direct effect on energy uptake and REE [89,90], and the beneficial role of exercise to delay these processes [91,92], only Australia and New Zealand developed a separate chapter [93]. Special guidelines regarding frailty in the geriatric population have been published for Europe [94], East-Asia [95], and Australia and New Zealand [96]. Most societies appreciate natural/hand feeding, and recommend oral supplementation, even for frail patients with severe cognitive decline [97]. As separate factors, present in the elder and frail individuals, such as mouth muscle tone loss, soft tissue trauma, coronal and dental root carries, low saliva flow, and osteoporosis may affect oral health, chemosensory perception, and feeding, researchers also proposed novel standards for the “ageing mouth” [98]. Overall, due to the multifactorial nature of energy and micronutrient imbalance in the elderly, any approach should be comprehensive [75]. At least the European Diabetes Working Party for Older People included evidence-based decisions in 9 clinical domains in its recent Position Statement [99].

Special attention is needed in cases of dementia, because of the apparent deficits in memory and verbal communication. Patients’ perception is rarely accurate in describing current and past dietary habits or weight change. Caregivers are the ones to conveniently report and handle any impairment from middle to late stages. Occasionally, they may miss gradual changes in appetite or weight, thus early opportunities for nutritional interventions [100,101]. The failure to report such events could be due to their adjustment to patient status. Most importantly, it appears that caregivers are also unable to recognize the symptoms in themselves, and may as well suffer from risk of malnutrition, or to a lesser degree from severe deficiencies [102]. The age of spouse or non-spouse caregiver could be an independent factor for malnutrition or frailty [69,78]. Considering the prevalence of deficiencies in the general adult population, younger individuals could suffer too for several reasons [67]. Subsequently, they would not make informed choices when assisting demented patients. In practice, it is advantageous for health professionals to perform a comprehensive and repetitive nutritional assessment, and employ biochemical markers.

## 6. To Grow or Not to Grow till Late Age?

Despite the lack of consensus, an array of tools permit the detailed quantitative and qualitative monitoring of nutritional status addressing perception [103], energy intake and expenditure [104], and post-absorptive nutritional adequacy [105]. Such inclusive approach though is costly and time-consuming. Recent data indicate that some biomarkers (or clusters) could be of potential value to assess compliance to, effectiveness, and risk of dietary patterns or interventions [106]. So far, there are no validated biomarkers to assess malnutrition and sarcopenia, while suitable molecules should be of longitudinal use, and capable to detect early metabolic or behavioural impairment, before gross (muscle) mass change takes place.

Basic research studies revealed that senescent cells share common molecular profiles with senescence accelerated mouse strains and accelerated ageing in humans [107,108]. Therefore, it would be preferable to adopt markers of biological rather than chronological ageing. The somatotropic axis appears the most convenient one, primarily due to its direct implication in development; ageing could be considered as its recession. Second, because of the functional role of growth hormone (GH) in anabolic processes, in both expansion and maintenance of fat-free mass, through direct and indirect mechanisms (e.g., somatomedins) [109,110,111]. These mechanisms also mediate separate actions, in peripheral and central level, in a variety of tissues [112,113,114], relevant to energy homeostasis (increased energy uptake, lipid mobilization, and mitochondria oxidation) and cell proliferation (DNA, RNA and protein synthesis, and enhanced cellular aminoacid uptake). Furthermore, the recently identified orexigenic peptide ghrelin and its agonists, known as growth hormone secretagogues, are promising agents in appetite and weight management in frail subjects [115,116]. Paradoxically, ghrelin autocrine signalling has been reported involved in the modulation of salty and sour recognition in ghrelin receptor null mice [117]. 

Early studies established that GH levels decline with age in both sexes, growth hormone-releasing hormone (GHRH) as well, and the effect is amplified when the effector Insulin-like growth factor 1 (IGF-1) is investigated [109,110,111]. Normally, hypoglycaemia and arginine trigger GH secretion, thus calorie restriction has been long believed an effective intervention to maintain higher GH and a young phenotype. Despite the induction of IGF-1, binding to Insulin-like growth factor-binding proteins (IGFBP) tempers its insulin-like properties under their mitogenic potency. IGFBPs remain quite constant with age, but their affinity is altered: reduced for IGF-1 [118], and increased for α2-macroglobulin dimers over monomers [119]. The decrease in hypothalamic catecholamines has been also linked to the reduction of somatotropic tone [120], and l-dopa administration to older men (*n* = 12, 78 ± 1 years old) resulted in lower GH and GHRH increments, as compared to young men (*n* = 12, 24 ± 1 years old) [121]. Age-related IGF-1 diminishment was prevented in older, but not young adult animals, by moderate dietary restriction, while protein synthesis or delay of decrease was enhanced in a tissue-specific way [122]. Similarly, a protein- and energy-deficient diet during development enhanced IGF-1 during adulthood specifically in kidneys [123]. Energy surplus, as estimated using somatometrics, was found an independent factor for IGF-1 decline during lifetime (17–83 years), significant in men, not in women [124], probably due to the differential dependency of somatotropes on leptin between sexes [125]. Furthermore, over the age of 50, IGF-1, IGFBP-3, and IGF-1:IGFBP-3 showed low intra-individual variability (*n* = 1618, 50–95 years old, 10 months–5.6 years follow-up), but the reduction of IGF-1 and IGFBP-3 was significantly broader in men, with IGFBP-3 decline being associated with comorbidities and decreased gait speed [126]. In women, IGF-1 and IGFBP-3 levels were associated with better performance in attention, visuospatial, and global cognitive tests, regardless of gait speed [127].

Because of the general decline of GH/IGF-1 with age, many attempts were made for the restitution of somatotropic signalling. GH administration in healthy elders showed contradictory results on substrate preference, lean mass:fat mass and the improvement of bone turnover [128,129], while benefits were recorded in underweight subjects with serum albumin <38 g/L [130]. It is probable that pharmacological interventions present advantages when functional impairment is vast. IGF-1, in contrast to adiponectin, IL-6 and cystatin-C, was not found to correlate with several functional declines indices (*n* = 3372, 73 ± 6 years old, 7 years follow-up) [131], but subjects within the lowest quartile of IGF-1 (*n* = 4133, 72 ± 6 years old) were more likely to exhibit pre-frailty or frailty [132]. On the other hand, population studies exploring single nucleotide polymorphisms (SNPs) involved in GH signalling were not able to detect relationships with physical or cognitive performance in two large cohorts of almost centenarian Danes [133], while animal models of GH deficiency exhibit remarkable longevity, reduced cell senescence and improved metabolic profile [134]. Therefore, there is also a possibility that the weakening of somatotropic axis in humans could bear protection over chronic conditions. If other environmental stressors were prevented, both length and quality of life could be maintained, as particularly seen in daf-2 *C. elegans* mutant, lacking insulin and IGF-1 receptor [135]. Still, this is no one’s story.

## 7. Energy Balance, Micronutrient Deficiencies and Adipokines in Dementia

Chronic metabolic conditions, especially glucose metabolism dysregulation, are currently in focus as for their causative or precipitating role in dementia [17]. Appetite and/or weight change though are hallmarks within the battery of BPSD [5,21,136]. As the severity of these measures varies during the course of dementia and among its different subtypes [8,11], it could be possible for subtle changes to be detected long before a diagnosis. Absolute weight at birth has been shown beneficial for cognitive performance in longitudinal studies at different developmental stages, while exposure to famine either during pregnancy or in early life has the opposite effect [137]. Weight gain, elevated weight and BMI during adulthood, especially peak adiposity in middle age (around 40–55 years), have also a detrimental effect in late cognitive life [138]. A recent meta-analysis of epidemiological studies with a minimum 2-year follow-up showed mixed results with an overall protective effect of obesity (BMI > 30) on intellect, and a 0.83 (95% CI: 0.74–0.94) relative risk of dementia incidence over 65 years [139].

Body composition is not usually assessed with uniform tools, and single measures of body conformation may result in reverse associations, as seen for abdominal adiposity and total adiposity or BMI [140,141,142]. A major drawback in the studies investigating BMI is also the use of height, the estimation of which may be problematic in the elderly, as well as the evaluation of somatometrics in fixed time points. A retrograde estimation of body mass, starting from the year of dementia diagnosis, counting back 5–6, 9–10, 11–20, 21–30, and >30 years, calculating median when multiple values were available, and using random age-matched control subjects free of possible symptoms within the next 3 years, showed that female, but not male, patients, exhibited significantly lower weight 20 years before incidence, and the odds of dementia increased over time for those within the lower weight quartiles [143].

Considering the common dietary patterns in community-dwelling elder adults, reduced consumption of all foods would increase the probability of dietary micronutrients’ deficiency [83], thus obesity could be of advantage. As for the case of homocysteine, folic acid, and vitamins B6 and B12, not only their separate estimation, but a misbalance among them has been implicated in the precipitation of dementia [144,145]. Moreover, impaired metabolism and low availability of some nutrients, as shown for vitamin E metabolites α- and γ-tocotrienol and γ-tocopherol, were found associated with mild cognitive impairment (MCI) and AD diagnosis, as well as with prospective regional atrophy in the medial temporal lobe (e.g., entorhinal cortex, fusiform gyrus, isthmus of cingulate cortex and middle temporal gyrus) and orbitofrontal cortex in MCI patients that developed AD 1 year later [146]. However, the improvement of cognitive status after the adoption of supplementation, or a diet with potential benefits on cardiovascular health or physical activity, depends on individual background and appears suboptimal in late life [147,148].

Adipocyte-derived hormones may mediate the effect of body composition on cognitive health, and lead to the discrepancies between sexes either prior or upon the manifestation of dementia. Adipokines play a major role in health and fertility, while adipocyte dysfunction and impaired adipokine signalling are observed in an array of metabolic and vascular disorders [149], also associated with dementia. Some authors reported higher peripheral adiponectin levels in MCI and AD in correlation with adiponectin in CSF [150]. Others reported an increase of adiponectin in AD or mixed dementia (MD) patients and of resistin in VD patients, regardless of the presence of abdominal obesity [151], or elevated, but not significant, adiponectin and increased reptin:adiponectin in MCI and AD, but not in VD [152]. Additionally, only in female participants of the Framingham Heart Study, adiponectin increase by 1 SD during the 13-year follow up correlated with 30% higher risk of all types of dementia or AD [153]. Other groups found no difference either for the low [154] or the high molecular weight (HMW) forms of adiponectin [155], while in the Osaka Follow-up Study for Carotid Atherosclerosis HMW-adiponectin was lower in all male participants than in females, and inversely correlated with BMI. On the contrary, some groups published decreasing levels of adiponectin, not sensitive to the decline of cognitive indexes though, as compared to control subjects [156], and a parallel increase in leptin, associated with BMI and HbA1c [157]. Specifically, HMW-adiponectin was found lower in a sub-group of AD patients with HbA1c ≥ 7% and impaired daily activities consequent to DM2; apathy, overeating and excessive daytime sleeping were prominent, despite the absence or other functional cognitive differences among diabetic and non-diabetic individuals [158].

The aforementioned variations in adipokines’ concentration may be due to divergent dietary patterns. In the Japanese Multi-Institutional Collaborative Cohort (*n* = 697, 35–69 years old), adiponectin levels were associated with consumption of bread and dairy products, and low intake of rice, and inversely correlated with waist circumference and insulin resistance, as estimated using the homeostasis model assessment (HOMA-IR) [159]. Another possibility is concurrent modifications of energy metabolism and muscle tone, because of the presence of atypical parkinsonian symptoms or cases among dementia patients [160], as well as the administration of acetylcholinesterase inhibitors (ACHEIs) [161]. Donepezil has been shown to attenuate muscle atrophy and induce neovascularization in vitro and in a murine hind limb ischemia model [162], and enhance neuronal mitochondrial biogenesis and oxidative phosphorylation in vitro and ex vivo [163], while it up-regulated adiponectin over leptin synthesis, towards a reduction of total and abdominal fat deposition, in stage 3 to 4 AD patients, within 6 months [164]. Galantamine-induced adiponectin augmentation and HOMA-IR amelioration have been reported so far in one study in young adults with metabolic syndrome within 4 weeks [165]. On the contrary, rivastigmine, capable also of inhibiting the ghrelin degrading enzyme butyrylcholine esterase, has a direct effect on appetite and the prevention of weight loss, without affecting leptin levels [166,167].

The interpretation of epidemiological data could substantially improve by the availability of tissue-specific data. A recent ex vivo study of AD patients’ fibroblasts revealed partial similarities to both young and old control subjects: AD fibroblasts (i) had more mitochondria than those from older individuals, lower though than young controls, (ii) were less sensitive to IGF-1, as old cells, (iii) had significantly lower nicotinamide adenine dinucleotide (NAD) content, (iv) of which the continuous de novo synthesis was probably compensated by the induction of nicotinamide mononucleotide adenylyltransferase 2, (v) had almost a double NAD^+^/NADH ratio, as compared to all controls, in accordance with increased glycolysis in basal conditions, as well as after challenge with the ATPase inhibitor oligomycin or the mitochondria uncoupler carbonilcyanide p-triflouromethoxyphenylhydrazone, and (vi) a young ROS profile [168]. Surprisingly, despite other enzymes of Krebs cycle being up-regulated, oxoglutarate dehydrogenase was down-regulated, as described previously in response to dietary protein imbalance [169]. Accordingly, Wang et al. showed that community-dwelling patients (*n* = 51) with mild-moderate AD consumed more energy per body mass, regardless of having lost weight or having appetite impairment, as compared to non-demented controls (*n* = 21) [101]. Nevertheless, serum cholesterol, haemoglobin, uric acid, transferrin, and fasting blood glucose were lower. Most importantly, 24-h urinary urea nitrogen was significantly lower (6.6 ± 4.1 vs. 9.0 ± 2.9 g), indicative of poor nutrient absorption. These data show prior adaptations occurring in real life, and accentuate the role of nutrients in cellular metabolism, whether they are consumed in disproportion, mal-absorbed or depleted.

## 8. Behavioural and Neurochemical Correlates of Appetite Impairment in Dementia

It is beyond doubt that dementia patients frequently develop particular eating and dietary habits and serious feeding difficulties [136], that could be considered as symptoms, epiphenomena of the neurodegeneration, or evidence of insufficient caregiving. The behavioural correlates of eating impairment are not uniform among the subtypes of dementia either prior to or after diagnosis. In VD patients, the prevalence of BPSD is as high as 90%, mostly associated with frontal injury [170]. In subcortical vascular dementia (sVD), half of VD cases, apathy is the most common symptom, followed by depression and agitation, while appetite impairment is less common. Among FTD cases, semantic primary progressive aphasia (sPPA) patients usually exhibit stereotypic behaviours, refuse to eat or consume very small amounts of food [171]. On the other hand, hyperphagia is prominent in behavioural variant FTD (bvFTD) patients. The severity and frequency of symptoms also varies through the course of disease. Eating disturbances are about 3 times higher in AD patients (*n* = 220) vs. non-dementia controls (*n* = 30), shifting from appetite change in mild AD towards sweeter preparations and higher use of condiments in moderate AD, encompassing swallowing problems in late stages [10,11]. Such transient changes may explain the differentiation of BMI before the final manifestation of weight loss [172]. Moreover, the symptoms emerge together with other components of NPI, particularly apathy, and have been shown to correlate with depression, euphoria and disinhibition in ambulant outpatients with moderate AD (*n* = 421) [13]. Disengagement and functional deterioration, as assessed with London Psychogeriatric Rating Scale, have been repeatedly associated with lower energy intake at lunch, and especially dinner, but not at breakfast in nursing home residents (*n* = 32, 88 ± 4 years old) [12]. Additionally, agitation, irritability, and disinhibition ratings were in line with increased carbohydrate selection over protein. The use of anorexic or orexigenic medication had no impact on total energy intake, such as there was a ceiling. Orexigenic medication though favoured carbohydrates over fat at breakfast, and protein intake at dinner. The latter effect could be of value, as options later in the day are the most unsatisfactory, regarding nutritional adequacy. Finally, a longitudinal study showed that acute episodes, related to appetite and weight disturbances, as seen in major depressive disorders, may further increase the hazard of AD by 69%, and of all dementia by 110%, with the risk of non-AD being higher when the first episodes occur earlier in life [60].

The differential behavioural impairment among dementia cases may be due to discrepancies of perceived valence, rather than of sensitivity, as discussed earlier. Sweetness perception, explored against desserts containing 26, 39, or 60% sucrose, was found similar among all subjects (N_bvFTD_ = 19, N_SPPA_ = 15, N_AD_ = 15, N_CONTROL_ = 25, age- and BMI-matched), but patients with bvFTD and sPPA expressed a strong preference for the sweetest dessert, while the bvFTD group disliked the least sweet [171]. Common neuronal correlates included volume loss in bilateral orbitofrontal cortices and right-sided insula-striatal structures, such as nucleus accumbens and amygdala, extending into the temporal occipital cortex, lingual gyrus, and cerebellum. When the same group was free to form breakfast choices, all dementia patients opted for lower protein intake, especially bvFTD patients. Total breakfast calories correlated with caregivers Appetite and Eating Habits Questionnaire (APEHQ) ratings, and were at least doubled in bvFTD. A possible contribution of visual information was discussed, as total caloric intake was associated with volume loss in the lateral occipital and lingual cortices, indicative of a feedback mechanism connected to reward circuits. Another study in bvFTD patients detected a significant progressive atrophy of the posterior hypothalamus antemortem and postmortem [173]. The loss was greater in patients with TAR DNA-binding protein 43 (TDP-43) pathology, but did not include hypocretin/orexin, neuropeptide Y, cocaine-and-amphetamine-regulated transcript (CART) and vasopressin-containing neurons. These findings suggest a loss of internal inhibitory regulation at the level of hypothalamus, that could possibly lead to hyperactivity of peptidergic pathways, a disruption also identified in attention-deficit/hyperactivity disorder with binge eating [174].

Orexins’ signalling may be also modified through their receptors and their heterodimerization. Post-mortem examination showed down-regulation of OX1R, OX2R, and GPR103 in cornu ammonis, and of OX1R in dentate gyrus in late onset AD, and of GPR103 in dentate gyrus in both late and early onset cases [175]. Although amyloid beta 42 (Aβ42) induced reduction of all these receptors’ expression in the neuroblastoma cell line SH-SY5Y, it is not clear whether in vivo down-regulation is due to Aβ, or the result of diminished circulating and localized orexin, consistent in most dementias, including AD [176,177,178]. Orexin deficits are predominantly related to the disruption of sleep-wake cycle, extending to day apathy, agitation and irritability, and finally food preference [179]. Brand new evidence on the cholinergic release of GABAergic inhibition on hypothalamic orexin neurons [180] is in agreement with the reported broad spectrum of ACHEIs’ actions.

## 9. The Emerging Role of Salience Networks in Appetite Regulation

The perspective pharmacological interventions in the sub-group of MCI cases whose deterioration is anticipated [2,181] could be beneficial in alleviating appetite/eating disturbances and providing tailored solutions, due to the divergent actions of ACHEIs [164,165,166,167]. The NMDA antagonist memantine has been also reported capable to mitigate overall NPI prevalence in combined schemes with donepezil, and especially up to 4.8 points in replacement schemes [182], with significant improvement of the appetite/eating cluster, possibly due to the normalization of binge-eating [183]. The parallel actions against the BPSD array are fortunate [184], as only timely use of anti-dementia medication is suggested at the moment [2]. However, with the exception of rivastigmine, little evidence exists about the pathways implicated in appetite regulation during these interventions. Thus, our knowledge about the initiation and progression of impairment remains yet limited.

Eating disturbances appear early in the course of dementia [185], while depressive and vegetative symptoms, contributing directly or indirectly to energy/micronutrient imbalance, and vice versa, are present in ageing subjects [6], and in most MCI cases regardless of a positive dementia diagnosis [3,186]. In clinical practice, at least the pharmacological management of depression is problematic, because of the established risk of MCI due to anticholinergic medication [187]. Simple modifications though in daily routine may have considerable impact on neuropsychiatric symptoms independently of disease stage [2], addressing even disregarded physiological causes, such as chewing difficulties [26]. Positive reinforcement could also be advantageous, as reduced attention during meals has been shown to associate with poor appetite, and Geriatric Depression Scale ratings, in both MCI and AD, and despite concurrent psychoactive medication in AD [184]. These findings emphasize the role of incipient anatomical, physiological and metabolic changes, as discussed in previous chapters, in the manifestation of several behavioural (epi)phenomena with malnutrition as final outcome. On this background, comorbidities, including dementias and DM2, could intervene in a polytomous manner.

The arising question is what locus or loci could possibly link food chemosensation with food seeking behaviours and simultaneously mediate extreme and divergent nutritionally relevant behaviours, present in normal ageing, as well as in accelerated cognitive decline. Following the initial psychological/physiological studies that addressed separately food modalities and their conceptual sub-components, recent studies employing neuroimaging in individuals with or without lesions proved that multisensory integration is necessary for the discrimination of food vs. non-food flavours, and the affective configural learning of stimuli, implicating insula, the overlying operculum, the orbitofrontal cortex, and the anterior cingulate cortex [188,189]. Insula processes various sensory information with strong subjective affective value, and determines the relative salience of stimuli to initiate learning [190]. Projections to prefrontal areas ensure the assimilation of salient experience into cognition and goal-oriented decisions, while those to cingulate cortex are involved in the recognition of error and the adaptation/re-programming of goal-directed behaviour [191]. In the elderly, the available stimuli do not usually overcome previously set thresholds, because of the debilitation of sensory perception and the tedious life. Therefore, compensatory behaviours fixating in past knowledge, such as rigidity or cravings for comfort food, especially sweets, may arise. Negative feelings, due to digestive discomfort or difficulties in swallowing, may further potentiate adaptive aversive behaviours, with serious impact on calorie intake [59]. Apparently, versatile non-pharmacological approaches that involve enhanced multimodal input, implicit suggestions, ease of discomfort and fun are effective in alleviating burden [2,63].

In dementia, localized neurodegeneration may distort or erase learned priorities and finally annihilate attention. Specifically, in FTD, functional connectivity within the salience network, assessed in resting state using functional magnetic resonance imaging, was found decreased, while the opposite has been reported sporadically for AD [192]. At the histological level, in FTD, the characteristic large bipolar von Economo neurons (VENs) of frontoinsular and anterior cingulate cortex were severely and selectively targeted, down to 90% in the left side [193,194]. Because of the outnumbered VENs in the right side, and the rest of population being unaffected, the density of the area was also reduced [171]. In AD and amnestic MCI, VENs’ neurodegeneration was correlated with Braak stage, but was less pronounced than in FTD [193,195]. These findings are in line with the prevalence of disinhibition, compulsory eating and pica since early FTD and mild-moderate AD. Surprisingly, “Super Agers”, subjects >80 years old, with episodic memory equal or better that the one of 20–30 years old, had higher VENs density, even when compared to young adults [195].

## 10. Conclusions

Biological senescence materializes as a deterioration of physiological systems, with severe impact on the recognition of exteroceptive or interoceptive stimuli, their processing, and (adaptive) responses. Specifically, peripheral loss of taste/odour receptors or neurons causes significant deficits in the perception of tastant’ dimensions, quality, intensity and valence. Second, weakening of somatotropic signalling resets energy partition among cell populations, substrate preference, and metabolism. It is also probable for the ageing body to misread satiety signals, with insulin resistance playing the dominant role in glucose neglect. Therefore, elderly may formulate adaptive behaviours to address shifts in cognition and metabolism that evolve gradually into quantifiable changes in appetite and eating and finally body composition. Several tools allow us to monitor macro- and micronutrients’ intake, absorption and availability, hormonal regulation, metabolic rate and fat vs. not fat mass. However, it is not clear yet what biomarkers would be satisfactory in predicting malnutrition and frailty, during which muscle mass and functionality are wasted. On the other hand, normal or accelerated neurodegeneration, due to diabetic complications and dementia, modify low- and high-order circuitries, involved in the perception of food modalities and food seeking behaviours. It is beyond doubt that cognitive impairment is also accompanied by eating disturbances, correlated with concurrent neuropsychiatric manifestations. Recent evidence from imaging studies supports the shift or scatter of individual attention towards multimodal stimuli over time, while the beneficial role of behavioural interventions and of limited pharmacotherapy on these clusters emphasizes the affective component of food intake. In line with the suggested early, tailored and unified approaches, addressing appetite/eating impairment as a nutritional imbalance with incentive value in a given social context would substantially help individuals to thrive in late life.
